# Predicting socio-economic levels of urban regions via offline and online indicators

**DOI:** 10.1371/journal.pone.0219058

**Published:** 2019-07-10

**Authors:** Yi Ren, Tong Xia, Yong Li, Xiang Chen

**Affiliations:** 1 School of Electronics and Information Technology, Sun Yat-sen University, Guangzhou, China; 2 Key Lab of EDA, Research Institute of Tsinghua University in Shenzhen (RITS), Shenzhen, China; 3 Department of Electronic Engineering, Tsinghua University, Beijing, China; University of Warwick, UNITED KINGDOM

## Abstract

Predicting the socio-economic level of an urban region is of great significance for governments and city managers when allocating resources and making decisions. However, the current approaches for estimating regional socio-economic levels heavily rely on census data, which demands significant effort in terms of time and money. With the ubiquitous usage of smart phones and the prevalence of mobile applications, massive amounts of data are generated by mobile networks that record people’s behaviors. In this paper, we propose a low-cost approach of using humans’ online and offline indicators to predict the socio-economic levels of urban regions. The results show that the socio-economic prediction model that is trained using online and offline features extracted from these data achieves a high accuracy over 85%. Notably, online features are showed to be tightly linked with socio-economic development. In environments where censuses are rarely held, our method provides an option for timely and accurate prediction of the economic status of urban regions.

## Introduction

The accurate and real-time prediction of the socio-economic level (SEL) of a region plays an important role in grasping the development level of the city’s region. The government makes its decisions regarding coordinating the overall development according to the distribution of SELs. The current methods to investigate SELs mainly rely on the economic census that is organized by the National Statistical Institute (NSI). The NSI organizes the economic census every five years in China, which makes it difficult to provide timely referential data. In addition, the NSI counts the Gross Domestic Product (GDP) every quarter, which incurs great manpower costs. Therefore, a relatively low-cost approach is needed to estimate the SELs of urban regions.

Studies about economics predictions [1–5] using novel datasets have been conducted for a few years. Previous works have used mobile phone data to evaluate socio-economic status [3, 6, 7]. Some studies [2, 8, 9] extracted social, behavioral and mobility features from calling details records and trained machine learning models based on these features. Luca Pappalardo et al. found that there is a tight correlation between the aggregated human mobility patterns that are discovered from mobile phone data and socio-economic indicators [10]. They computed the mobility volume and mobility diversity at the individual level and aggregated them at the municipality level. Ultimately, they indicated that the aggregated mobility measures are correlated with socio-economic indicators. However, due to the expansion of the Internet age, people use chat applications increasingly more frequently instead of making calls. Therefore, methods that use the features that are extracted from calling data to predict socio-economic levels may become less useful. Other researchers attempted to identify economic indicators using satellite imagery [11, 12] or night time light [4]. However, this prediction would be outdated due to the popularity of the infrastructure. In wealthy countries, the data that are collected from the Internet and social media have been used to evaluate SELs [13, 14]. For instance, studies have proposed approaches that utilize Google Trends to predict short-term values of economic indicators [15] and unemployment benefits [16]. Some other previous works showed that social networks [17–19] have greatly impacted economics.

Existing research concentrates on using the mobility features that are extracted from calling detail records to predict economic status. However, with the popularity of the mobile Internet and widespread usage of smart phones, applications are bleeding into daily life and calls are gradually being replaced. Thus, the online features that are generated from application (App) usage records provide more valuable information than mobility features since these emerging Apps heavily influence people’s lifestyles, such as their shopping, communication and eduction. Some previous studies found that there are substantial differences in the App usage patterns for different subscribers [20, 21] and discovered that Apps usage can reflect people’s behaviors and preferred lifestyles [22]. Another study differentiated mobile phone users according to their used Apps [23]. According to this information, we expect that the online indicators that are extracted from Apps usage data should be associated with SELs. In addition, another study [24] discovered the functions of city regions according to human mobility and points of interests (POI), which inspires us to investigate whether the distribution of the POIs in a region may be an important indicator of SELs.

In this paper, we propose a novel approach to estimate SELs of blocks in Shanghai using the online and offline features of an urban region. The online and offline features that are extracted from App usage records and the distribution of the POIs in the region are entered into Random Forests (RF) or support vector machines (SVM) in order to predict SELs of blocks in Shanghai. Our approach achieves better performance due to considering the online features compared with previous work that just considered offline features. We find that frequent mobility corresponds to developed economic status. In addition, the area with a large quantity of *finance* POIs tends to be in high socio-economic levels. Furthermore, we find a tight correlation between App usage patterns and economic levels. People in wealthy areas are more active on networks and spend more time on Apps in travel category. This method provides a more precise approach to classification prediction than previous methods using calling details data [8, 9], which achieved the 86.6% accuracy for 3 SELs and 72.7% accuracy for 4 SELs in the blocks of Shanghai. In addition, it addresses the problem of high manpower and time costs when NSI estimates the SELs of regions. Our research provides reliable and timely predictions for policy makers to allocate resources for regions with different SELs, which can promote the equality of economic growth and improve people’s livelihoods.

## Materials and methods

### Datasets

Shanghai is a metropolis in China that is known as a world-famous financial center. It is composed of 16 districts and divided into 9858 regions according to the coverage of the base stations. However, the base station level is extremely fine-grained. In addition, we cannot obtain the GDP at the base station level. Thus, we investigate the SELs of the urban regions in Shanghai at a coarser granularity and aggregate 9858 base stations into 188 blocks according to their location information. The block is an administrative area lower than a district. The Shanghai government divides the 16 districts into 215 blocks for administrative ease. Our datasets cover 188 blocks in 15 districts except for the Chongming district. The boundary of each block consists of discrete points with longitudes and latitudes. Comparing the base station location data to the boundary location data of blocks, we assign the 9858 base station regions into 188 blocks. We use POIs data, App usage records and the GDPs of the urban regions in the SEL prediction. For each block in Shanghai, the features that are entered into prediction model contain over 6000 online and offline features, including App features, flow features, POI features and origin-destination (OD) features. In next section, we illustrate how to extract features for blocks from App usage records and POIs data.

#### POIs data

We obtained POIs in Shanghai which refer to locations that are associated with certain functions such as companies, restaurants, schools, government agencies, etc. We crawled the 782,528 POIs of Shanghai from Baidu Map service and complied with the terms and conditions of the platform. According to the functions of POIs, we divide the POIs into 21 categories. There are *restaurant*, *hotel*, *shopping* *mall*, *entertainment*, *industry*, *fitness*, *office* *building*, *residence*, *view* *spot*, *travel*, *education*, *finance*, *company*, *factory*, *life* *service*, *technology* *park*, *economic* *development* *zone*, *high* − *tech* *development* *zone*, *town*, *business* and *village*. The POIs data include 9,858 records. Each record consists of the base station ID and the number of each POI category within the coverage of the base station. The POI distribution reflects the offline characteristics of the regions.

#### App usage records

We obtained a historical log of anonymous App usage records in Shanghai from China Telecom, which is one of the major mobile operators in China. Our dataset contains anonymous cellular data access traces that were obtained using Deep Packet Inspection (DPI) appliances. DPI is a popular approach that is used for accurately detecting traffic in terms of the content, Apps, and individual users [25]. After DPI, each access log to the cellular network is characterized by an anonymous user ID, timestamp, cellular base station with GPS location and connection metadata. The ethical issues of the data have been considered carefully. All the App usage records that are used in our research are anonymized. In addition, all the datasets are stored in a server that is not connected to the Internet. Furthermore, all the researchers that participate in this work signed a Non-Disclosure Agreement and agree not to use the data for any other purpose. More details about how to obtain data from China Telecom and how to protect user privacy are illustrated in the S1 File.

The App usage records cover 15 districts of Shanghai, with the exception of the Chongming district. The data set contains the records of 20,000 subscribers over a period of one week from April 20th to April 26th, 2016. The logs contain 2,000 unique Apps and cover 9,858 base stations. Each log has the form of a tuple of the user ID, timestamp, base station ID, and App ID. The records with spatio-temporal information can be used to reveal human mobility patterns. With the locations of the base stations that a user went through, the user’s mobility can be generated as offline features. Moreover, the App usage records reflect users’ online characteristics [22, 26, 27]. We can observe the online features, including the category of the and the duration use.

#### GDP of urban regions

To validate the prediction model, the ground truth data are obtained from the *Shanghai* *Economy* *Almanac* (2017) [28] that was edited by the Development Research Center of Shanghai Municipal People’s Government and The Sixth National Census. The almanac is the most systematic, complete and authoritative reference book that records the economic and social development of Shanghai. From this book, we obtained the Gross Domestic Product (GDPs) of 15 administrative districts of Shanghai in 2016. Moreover, we obtained the populations of the 188 blocks from The Sixth National Census. To achieve a fine-grained prediction of the regions in Shanghai, we computed the GDPs of the blocks using the GDPs of the 15 administrative districts and populations of 188 blocks. The calculation formula is displayed as follows:

$$\begin{array}{c} G_{b}=G_{d}P_{b}/P_{d} \end{array}$$ (1)

where *G*_*b*_ represents the GDP of a block and *G*_*d*_ represents the GDP of the district to which the block belongs, and *P*_*b*_ and *P*_*d*_ are populations of district and block, respectively.

With the GDPs of the 188 blocks in Shanghai, we calculate the average GDP of the blocks and define 3 SELs (*A*,*B*,*C*) and 4 SELs (*A*,*B*,*C*,*D*), respectively, according to the difference between the GDPs of the blocks and the average GDP, where A represents the highest socio-economic level. The GDPs of the 188 blocks range from 21.75 to 671.11 with an average of 142.66. For the 5th block lacks POIs data, we drop it when training the model. For 3 classes, *A* covers the range [100 ∼ 300), *B* covers [−100 ∼ 100) and *C* covers [−300 ∼ −100). For 4 classes, *A* covers the range [100 ∼ 300), *B* covers [0 ∼ 100), *C* covers [−100 ∼ 0) and *D* covers [−300 ∼ −100). There are a few blocks whose GDPs are higher than all the defined SELs. We just assign them to the highest level. The distribution of blocks across 3 classes is 26 blocks in level *A*, 148 blocks in level *B* and 13 blocks in level *C*. The distribution of blocks across 4 classes is 26 blocks in level *A*, 39 blocks in level *B*, 109 blocks in level *C* and 13 blocks in level *D*.

### Feature engineering

The objective of feature engineering is to generate indicators that can be used to predict the SELs of regions. In the existing work, the mobility features are regarded as effective indicators for predicting socio-economic status. In our work, we seek to explore more effective indicators of SELs. Apart from mobility features [24, 29, 30] such as the flow features and OD features, we extract the POI features and App features from row POIs data and App usage records. The offline features consist of POI features, flow features and OD features, while the online features represent the App features. In this paper, we extract the features that are mentioned above from App usage records and the POIs data at the block level and enter them into the RF or SVM to predict SELs of the blocks in Shanghai.

#### Baseline models: Mobility features, offline features and random classifier

In addition to the model we proposed below, we introduce three baselines for comparison purposes. The first baseline is a mobility based model, which consists of the 5 most important flow features and the 5 most important OD features. The second baseline is a model using offline features, which consists of the mobility features that were used above and the 10 most important POI features. The third baseline is a random classifier whose features are the same as our model’s features. The difference between random classifier and our model is that the training sets of random classifier are chose randomly. Since the distribution of blocks at different SELs is unbalanced, we use cost-sensitive to solve this problem in our model. Meanwhile, in the random classifier, it chooses the training samples from each class randomly. Next, we illustrate how to extract these features and evaluate the importance of features using formula (2).

Jing Yuan et al. found that the functions of different city regions are correlated with the POIs in them. We expect to find that the distribution of the POIs within blocks is effective for predicting SELs. We then aggregate the POI distribution of the base stations at the block level by comparing the locations of the base stations with the boundaries of each block. Since the 5th block lacks POI data, we exclude it and assigned the 9858 base station records to 187 blocks. Ultimately, for each block, the POI feature is a vector consisting of number of POIs belonging to the 21 POI categories.

The flow features represent the incoming and outgoing visitor flowrates of the regions. We assign the 9,858 base stations to 188 blocks and observe the flowrate of these blocks during 8 three-hour periods. To avoid the situation that the subscribers just pass through a location but do not stay for long, we set a time threshold Δ*T* to define whether they stay. In our research, we set Δ*T* = 30 minutes according to the experience of previous work [31]. Only when the user stayed in a block for a time greater than Δ*T* can the block be treated as an effective stay point. Thus, the flow feature vector of each block is represented by a 2 (incoming and outgoing)×2 (weekdays and holidays)×8 (time periods)vector whose element represents the average daily flowrate at a certain period. For instance, once an individual leaves stay point *j* at AM 8:00 and arrives at stay point *i* at AM 10:00 on Sunday, a value of 1 will be added at *OUT*_*H*06 − 09 of block *j* and *IN*_*H*09 − 12 of block *i*, respectively.

We observed transitions between the original block and destination block as the OD features. We defined the OD features as transitions where people go to or leave a block during a certain period [9]. For instance, given two App usage records of the same subscriber, an OD feature can be denoted as follows. If a subscriber leaves stay point j at AM 8:00 and arrives at stay point i at AM 10:00 on Sunday, a value of 1 will be added at *OUT*_*i*_*H*06 − 09 of block *j* and *IN*_*j*_*H*09 − 12 of block *i*, respectively. Ultimately, the OD feature of a block is represented by a 2 (incoming and outgoing)×2 (weekdays and holidays)×8 (time periods)×188 (origination or destination) average mobility motif tuple.

#### Our model

Our model combines all the offline features that are mentioned above and the App features. We then input them into the machine learning algorithms in order to train the prediction model. Apps that people pay attention to vary according to their role(s). For example, student groups may spend more time on education Apps, while white-collars may pay more attention to financial Apps. Thus, we assume that the App usage preferences of the people in a region may have some correlations with the SELs of the region. The App features are extracted from App usage records as follows.

There are 2,000 unique Apps in the App usage records. The number of Apps is extremely large and some of the Apps have similar functions. Thus, the Apps are divided into 18 categories, as shown in Table 1, according to their core functions, which are provided by the App store. The motivation for why we extract App features is that App usage can reflect users’ behaviors and preference. However, the two categories of *System*_*tool* and *Others* cannot provide valuable feedback. Therefore, we drop these two categories when we extract the App features. In order to further observe the influence of different time periods on App usage, we separate a week into weekdays and holidays. Each day is divided into 8 three-hour periods i.e., *T* ∈ {[0 ∼ 3),[3 ∼ 6),…[21 ∼ 24)}. For each block, the App feature is represented by a 2 (weekdays and holidays)×8 (time periods)×16 (App categories) vector. For example, the element in *financial*_*H*12 − 15 of block *i* reflects the average usage duration of the *financial* Apps in the 12:00-15:00 PM period on holidays in block *i*.

**Table 1:** App category.

App Category	Sample Apps	App Category	Sample Apps
Social	QQ, Wechat	Finance	Alipay, Ant fortune
Video	QIYIVideo, Pear Video	Transportation	Uber, Didi
Audio	QQMusic, QingtingFM	Navigation	Amap, Baidumap
News	SohuNews, QQNews	Life	Clouds weather
Browser	UC Browser	Travel	Qunar, Fliggy
Game	Happyelement	Office	Iciba, QQMail
Health	Keep, Yuedongli	Stock	Eastmoney
Shopping	Taobao, JD	System tool	WiFi, SogouInput
Ordering	Meituan, Eleme	Others	Androidasync

## Results

### Accuracy of prediction

#### Accuracy of prediction model

**Fig 1:**
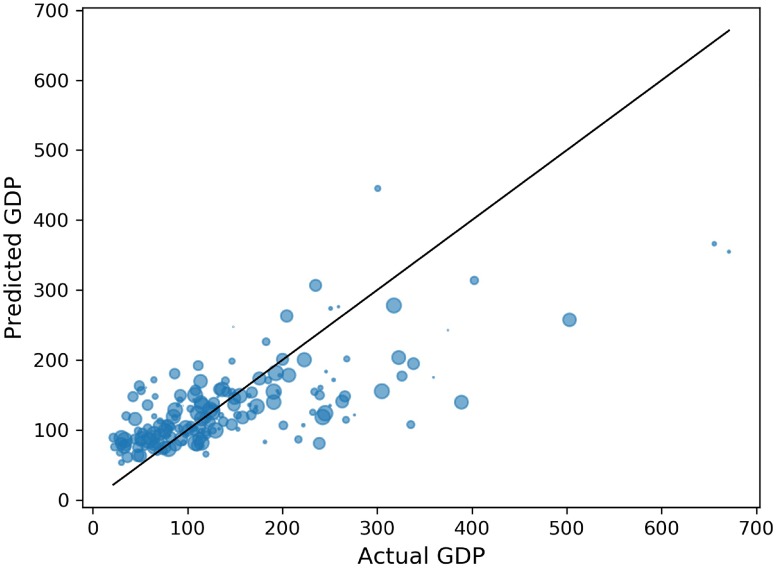
Comparison of GDP predictions to actual GDP.

The GDPs of the 188 blocks ranged from 21.75 to 671.11 with an average of 142.66. For the 5th block lacks POIs data, we drop it when training the model. We divide the 187 blocks into 5 different training and testing sets through 5-fold cross-validation. We fit a regression algorithm using the Random Forest to each set and obtain the average *R*^2^ of 0.474 as shown in Fig 1, which indicates that there is correlation between features that are extracted above and the socio-economic indicators. Since the previous works usually solve the economic prediction as classification problems, we will further analyze the results of the classification models.

**Fig 2:**
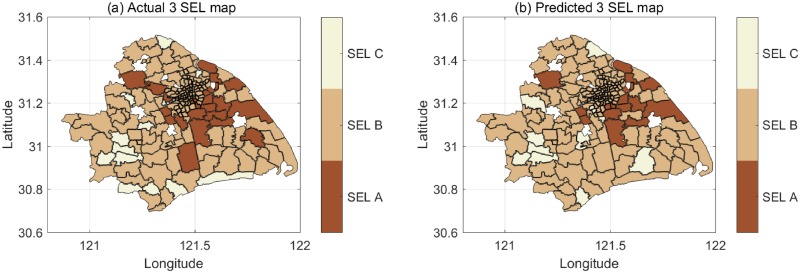
The actual and predicted socio-economic map of 3 SELs. a: Actual 3 SEL map. b: Predicted 3 SEL map with the accuracy of 85%.

**Fig 3:**
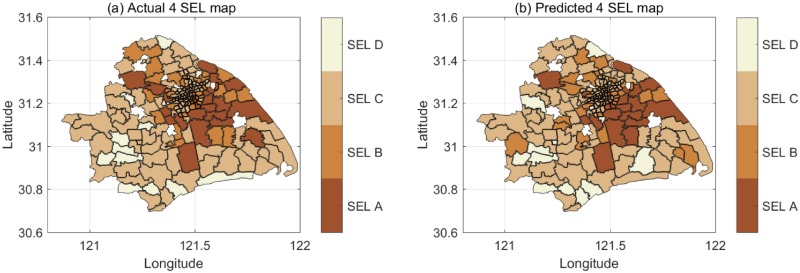
The actual and predicted socio-economic map of 4 SELs. a: Actual 4 SEL map. b: Predicted 4 SEL map with the accuracy of 64%.

Figs 2 and 3 show the socio-economic maps when classifying 187 blocks into 3 SELs and 4 SELs, respectively, in Shanghai. Fig 2(a) and 2(b) are the distributions of the actual SELs and the predicted SELs, respectively, with an accuracy of 85% for the 3 classes. Fig 3(a) and 3(b) are respectively the distributions of the actual SELs and the predicted SELs with an accuracy of 64% for the 4 classes. The darker shades represent richer areas. The blocks without a filling color lack data. From the actual SEL map, we can observe that classification of SELs is unbalanced. Thus, we apply cost-sensitive training when we train the models. The predicted SEL maps are obtained using SVM model through a 5-fold cross-validation. The model has good performance though the classification is unbalanced.

#### Comparison with baselines

We use 5-fold cross-validation to divide the dataset into 5 training and testing sets with an 80% − 20% split. We fit a RF or SVM on each training set and observe the model’s performance on the testing sets. Tables 2 and 3 show the accuracy (ACC), precision (P), recall (R) and F1 scores of different models for the 3 SELs and 4 SELs, respectively. For both the 3 classes and 4 classes, the trends of the performance indicators are similar. Compared with model using mobility features, the offline feature based model has approximately 4% better accuracy for the 3 SELs and approximately 3% better accuracy for the 4 SELs when using RF. Furthermore, the random classifiers evidently enhance the overall performance both with 3 SELs and 4SELs. However, the random classifier for the 3 SELs performs badly when predicting the blocks in SEL *C*, which is influenced by the unbalanced classes. Our models make up for this limitation to a certain extent and perform best on all aspects. In the case of our method, the highest accuracies for the 3 SELs and 4 SELs are obtained by using the SVM, while the RF models work better on detecting the blocks of SEL *C* or SEL *D*. The results indicate that POI features and App features play important parts in enhancing predictive performance.

**Table 2:** Accuracy, precision, recall and F1-score of different models for 3 SELs.

		SEL *A*	SEL *B*	SEL *C*	Total
		P/R/F1	P/R/F1	P/R/F1	ACC/P/R/F1
Mobility features	RF	0.57/0.31/0.40	0.82/0.93/0.87	0.00/0.00/0.00	0.781/0.73/0.78/0.74
	SVM	0.44/0.54/0.48	0.87/0.49/0.63	0.13/0.69/0.21	0.513/0.76/0.51/0.58
Offline features	RF	0.67/0.38/0.49	0.83/0.97/0.89	0.00/0.00/0.00	0.818/0.75/0.82/0.78
	SVM	0.30/0.54/0.39	0.85/0.55/0.67	0.22/0.77/0.34	0.561/0.73/0.56/0.61
Random classifier	RF	0.80/0.46/0.59	0.84/0.98/0.91	0.00/0.00/0.00	0.839/0.78/0.84/0.80
	SVM	1.00/0.23/0.38	0.82/0.89/0.85	0.23/0.38/0.29	0.759/0.81/0.76/0.75
Our model	RF	**0.91/0.38/0.54**	**0.84/0.99/0.91**	**1.00/0.08/0.14**	**0.845/0.86/0.84/0.81**
	SVM	**1.00/0.58/0.73**	**0.86/0.99/0.92**	**0.33/0.08/0.12**	**0.866/0.85/0.87/0.84**

**Table 3:** Accuracy, precision, recall and F1-score of different models for 4 SELs.

		SEL *A*	SEL *B*	SEL *C*	SEL *D*	Total
		P/R/F1	P/R/F1	P/R/F1	P/R/F1	ACC/P/R/F1
Mobility features	RF	0.43/0.35/0.38	0.17/0.08/0.11	0.61/0.81/0.70	0.25/0.08/0.12	0.540/0.47/0.54/0.49
	SVM	0.48/0.54/0.51	0.28/0.33/0.31	0.65/0.28/0.39	0.14/0.69/0.23	0.353/0.52/0.35/0.38
Offline features	RF	0.59/0.38/0.47	0.18/0.08/0.11	0.63/0.86/0.73	0.00/0.00/0.00	0.572/0.48/0.57/0.51
	SVM	0.38/0.46/0.41	0.32/0.31/0.31	0.72/0.46/0.56	0.23/0.85/0.36	0.455/0.56/0.45/0.48
Random classifier	RF	0.69/0.42/0.52	0.41/0.23/0.30	0.67/0.89/0.76	0.25/0.08/0.12	0.631/0.59/0.63/0.59
	SVM	0.89/0.31/0.46	0.38/0.15/0.22	0.67/0.83/0.74	0.21/0.46/0.29	0.588/0.61/0.59/0.52
Our model	RF	**0.81/0.50/0.62**	**0.41/0.23/0.30**	**0.68/0.92/0.78**	**0.33/0.08/0.12**	**0.657/0.62/0.66/0.61**
	SVM	**0.89/0.31/0.46**	**0.68/0.33/0.45**	**0.68/0.96/0.80**	**0.20/0.08/0.11**	**0.727/0.68/0.68/0.63**

#### Accuracy versus the number of features

**Fig 4:**
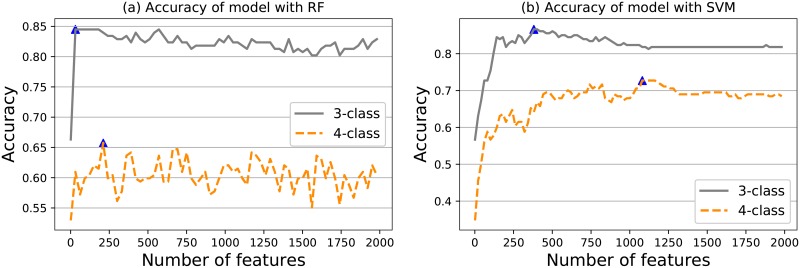
The accuracy of prediction model with RF and SVM. a: Accuracy versus number of features for RF. b: Accuracy versus number of features for SVM.

To further investigate how the model performance is affected by the number of features that is entered into the model, we compute the accuracy using 5-fold cross-validation for each subset of ordered features using RF and SVM. The ordered features are selected according to the ANOVA F-value of the samples. As shown in Fig 4(a), we observe that the RF for the 3 SELs achieves an accuracy of 84.5% when using the top 31 features, while the RF for the 4 SELs reaches 65.7% when using the top 211 features. The classification results using the SVM are presented in Fig 4(b). The SVM model for the 3 SELs achieves the best result, reaching an accuracy of up to 86.6% by using the top 381 features, while the model for the 4 SELs achieves the highest accuracy of 72.7% for the top 1081 features. Thus, the SVM outperforms the RF both for 3 classes and 4 classes.

### Comparisons between rich and poor areas

**Fig 5:**
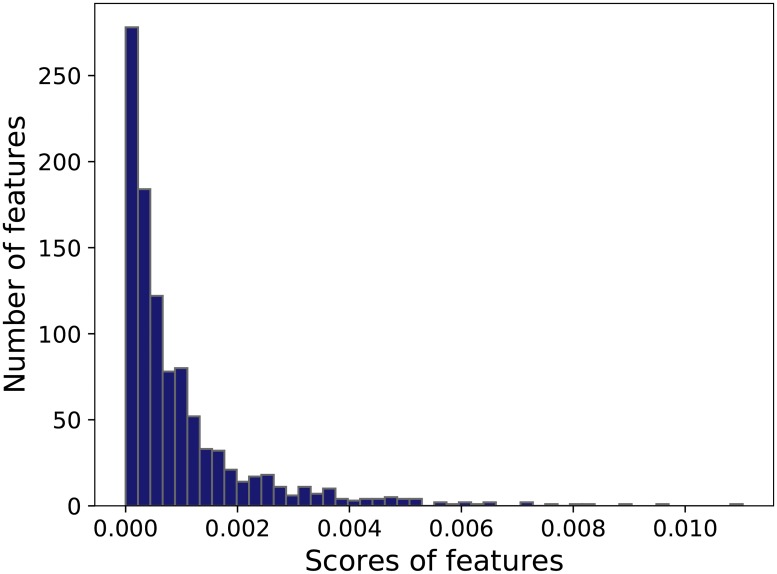
The distribution of feature scores.

We compute the importances of all the features when using the RF model and obtain 1018 features whose scores are greater than 0. The importance of feature *x* is defined as follows:

$$\begin{array}{c} I\left(x\right)=E_{o}\left(g\right)-E_{o}^{\left(n\right)}\left(g\right) \end{array}$$ (2)

where *E*_*o*_(*g*) is the out of bag (OOB) error of the RF model, while the $E_{o}^{\left(n\right)}\left(g\right)$ is the new OOB error when the values of feature *x* are inserted noise. The higher importance value of the feature is, the more important is the feature. The distribution of the feature scores is presented in Fig 5, which shows that the features with high scores just account for a small proportion. Table 4 shows the top 20 important features according to their categories and their scores. As shown in Table 4, the online features, flow features and POI features of offline features have significant importance, and we simply compare these features between rich areas and poor areas.

**Table 4:** Top 20 important features for the 3 classes using the RF. The online features, POI features and flow features of the offline features play key roles in predicting SELs.

Feature	Score	Feature	Score
IN_188_W15-18	0.011	Transportation_W09-12	0.0063
Navigation_W21-24	0.0096	Social_H21-24	0.0061
Finance POIs	0.009	Browser_H15-18	0.006
Browser_W21-24	0.0083	IN_H18-21	0.0059
Navigation_W06-09	0.0081	IN_H21-24	0.0056
Education POIs	0.0075	IN_W15-18	0.0055
Social_W18-21	0.0072	News_W12-15	0.0052
Office building POIs	0.0071	Browser_H12-15	0.0051
IN_W09-12	0.0066	OUT_W12-15	0.0051
OUT_H18-21	0.0064	IN_W21-24	0.0051

To further explore the correlation between the features and SELs, we selected the 5 richest and 5 poorest blocks in Shanghai. The rich blocks are administrative centers or economic parks, while the poor blocks mainly rely on agriculture. For convenience and to provide a clear illustration, we compute the average of the selected features of rich blocks and the poor blocks, respectively, and make comparisons.

#### The comparison of offline features

**Fig 6:**
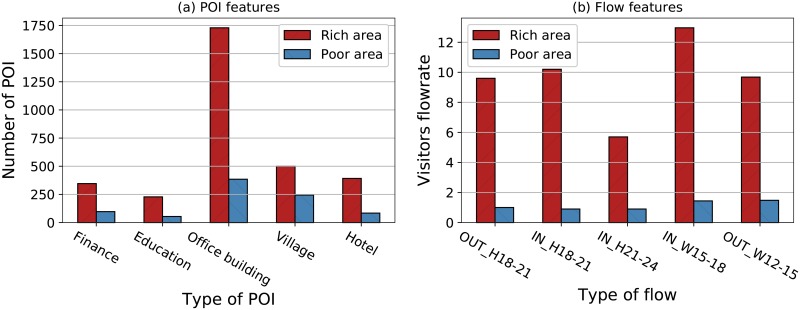
The comparison of offline features between rich areas and poor areas. a: POI features. b: Flow features.

As revealed in Table 4, there are 3 POI features in the top 10 features, which indicates that POI features have good correlations with SELs. For each block, the POI feature is a vector consisting of the number of 21 categories of POIs. We select the 5 most important POI features and make a comparison between rich areas and poor areas. The differences in the POI features between rich areas and poor areas are shown as Fig 6(a).

Apparently, the *finance* POIs have the closest correlation with economics. The gap between rich areas and poor areas with respect to *finance* POIs demonstrates that there are more financial business requirements for people in rich areas, from which we can infer that regions with more financial POIs may have higher socio-economic level. Moreover, more *education* and *office* *building* POIs are located in rich areas compared with poor areas. Therefore, more opportunities for learning and work are provided in richer areas. In addition, the difference between rich areas and poor areas with respect to *hotel* POIs is apparent, which indicates that the people visiting a city are more likely to stay in the richer area of the city. What is contrary to our expectations is that the rich areas have more villages. That may be because Shanghai is a highly urbanized city whose villages are not dominated by traditional agriculture. Thus, the large number of villages corresponds to the population to some extent. Areas with larger populations are more likely to be well developed. Apparently, the significant differences in the numbers of POIs between rich areas and poor areas correspond to the differences in the SELs. Thus, the categories of POIs are valuable indicators to socioeconomic levels.

For each block we investigated, the flow feature is a vector whose element represents the average incoming or outgoing visitor flowrate during a given period. Fig 6(b) shows the differences between rich areas and poor areas for the 5 most important flow features. From this comparison, we can observe that more people come in or go out on holiday evenings in rich areas, which indicates that people in rich areas actively engage in activities in the evening. For instance, people are likely to go to rich area for shopping, entertainment or leisure on holidays. In addition, on workdays, more people flow out during the 12:00-15:00 PM period and flow in during the 15:00-18:00 PM period. This phenomenon reflects the fact that people in rich areas have more career opportunities. In brief, the inflows and outflows of a region during a given period can accurately reflect the social economic levels to a great extent.Some other researchers also have found that people’s mobility is tightly linked with their economic status [32, 33].

#### The comparison of online features

**Fig 7:**
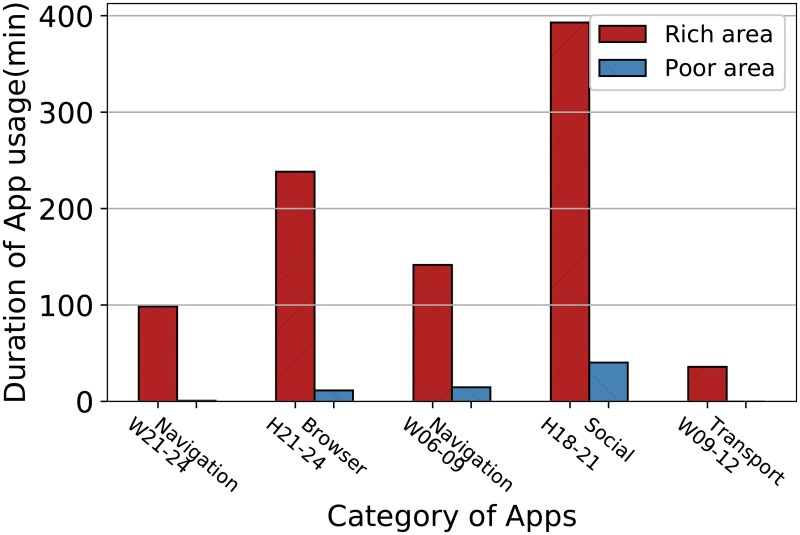
The comparison of App features between rich areas and poor areas.

Online features represent App features. Therefore, we select the top 5 most important App features and make a comparison between rich areas and poor areas. For each block, the App feature is a vector in which element is the average usage duration of a category of Apps during a given period in the block. The results in Fig 7 show the distinction between rich and poor areas.

As observed in Fig 7, people in rich areas primarily use *navigation* and *transportation* Apps, especially in the mornings and evenings on workdays. That could be caused by the fact that there are more working persons using *navigation* and *transportation* Apps on their way to work in rich areas. Accordingly, areas with larger workforces correspond to higher GDPs. In addition, on holiday evenings, people in rich areas spend more time on *social* and *browser* Apps, which indicates that they are more active on the Internet. The significant differences between rich areas and poor areas shows that these features are highly indicative of the SELs.

**Fig 8:**
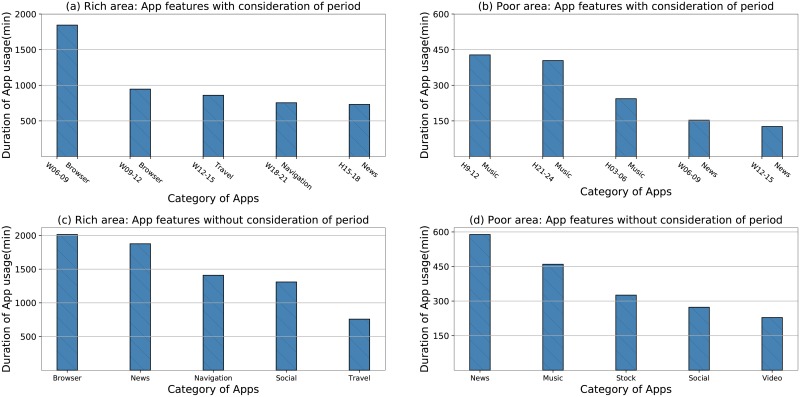
The 5 most frequently used App categories in rich areas and poor areas. a and b are App features with consideration of period. c and d are App features without consideration of period.

To further investigate the correlation between App usage and SEL, we select the 5 most frequently used App categories in rich areas and poor areas, respectively, as shown in Fig 8. Comparing Fig 8(a) with 8(b), we can find that the App usage categories are more diverse for people in rich areas. People in rich areas spend more time on *travel* and *navigation* Apps, from which we can infer that people in rich areas may engage in more public activities and they pay more attention to the quality of life. Conversely, people in poor areas primarily use *music* and *news* Apps. Their hobbies are relatively monotonous compared with people in rich areas. Furthermore, we explore the influence brought by App usage without considering the different periods, as shown in Fig 8(c) and 8(d). Comparing Fig 8(c) with 8(d), we find that people in rich areas pay more frequent attention to networking Apps. Simultaneously, they spend more time on Apps that are required when taking trips. Thus, Apps related to taking trips are strong indicators to economic status. Fig 8(d) shows that people in poor areas get their entertainment usually through *music* and *video* Apps. *Social* and *browser* Apps are also popular with people in poor areas, but the time they spent on these types of Apps is truly shorter than that of people in rich areas.

## Discussion and conclusion

The model we proposed performs better than the three simple baselines. The POI features and App features are showed to be tightly linked to economic status. We select some features according to their importance and analyze them. We find that POIs are good indicator to SELs. Areas with more *finance*, *education* and *office* *building* POIs are more likely to be of good economic status. Understandably, areas with more numerous opportunities and functions are likely to achieve high GDPs. Moreover, the App features reveal the fact that regions in which people frequently use Apps about taking trips during rush hour are more economically developed. Corresponding to the App features, areas with more people flowing in and out usually have high socio-economic levels, especially during rush hour and weekend evenings. This result reaffirms the rule that the areas with denser flows correspond to high socio-economic levels. Although our research provides a novel method to investigate the socio-economic levels, there are still some limits. It is difficult to collect App usage records in the areas where smartphones are not ubiquitous. In addition, our method has an inherent limitation in that it does not capture smartphone Apps that are used through WiFi networks. Thus, the online features are not compete to a certain extent. Our method may provide some inspirations for researchers who are investigating the Internet data and socio-economic problems.

## Supporting information

S1 File Data description and construction. Supplementary text for this article.
(PDF)
