# Supplementary material for: Predicting socio-economic levels of urban regions via offline and online indicators
Source: PLoS One. 2019 Jul 10;14(7):e0219058. doi: 10.1371/journal.pone.0219058 (PMC6619744; doi:10.1371/journal.pone.0219058)
Supplement: S1 File — Supplementary text for this article. (PDF) [file pone.0219058.s001.pdf]

Supporting Information for "Predicting Socio-economic Levels of Urban Regions Via Offline and Online Indicators"

A. POIs data

POIs are locations associated with certain functions such as companies, restaurants, schools, government agencies, etc. We crawled the 782,528 POIs of Shanghai from Baidu Map service (Baidu Map API Platform. http://lbsyun.baidu.com/). We divide the POIs into 21 categories by referring to the classification rule provided by Baidu Map (http://lbsyun.baidu.com/index.php?title=lbscloud/poitags). The categories are shown as follows, *restaurant, hotel, shopping mall, entertainment, fitness, school, scenic spot, tourism, finance, office building, company, business, factory, industry, scientific park, economic development zone, high-tech development zone, resident, life service, town, village.*

B. App usage records

The dataset contains anonymized cellular data accessing traces obtained by Deep Packet Inspection (DPI) appliances. Data was collected from mobile cellular network in Shanghai, one of the major metropolitan areas in China. Data requests on the network are passively inspected and captured. After deep packet inspection, each access record is characterized by an anonymous user ID, timestamp, cellular base station with GPS location and the metadata of the connection. We utilize the spatio-temporal information in this dataset as the mobility data to extract offline activities. To extract the online Apps usage, we adopt SAMPLES to identify the Apps from the HTTP header in the metadata.

Overall, we obtain the dataset containing over 1,700,000 unique devices, 2000 unique Apps, under 9800 base stations overall Shanghai with a period of 7 days from April 20th to 26th, 2016. We also divide these Apps into 18 categories by referring to the App Store (IOS Apps) and Google Play (Android Apps).

SAMPLES: *Hongyi Yao, Gyan Ranjan, Alok Tongaonkar, Yong Liao, and Zhuoqing Morley Mao. Samples: Self adaptive mining of persistent lexical snippets for classifying mobile application traffic. In Proceedings of the 21st Annual International Conference on Mobile Computing and Networking, MobiCom '15, pages 439–451, New York, NY, USA, 2015. ACM*

We have carefully considered the ethical issues of the data and taken effective measures to protect user privacy. First, when the users use the network service provided by the cellular network operator, they have authorized that their data can be collected and analyzed by the provider. Moreover, all the personal identification information have been stripped (or replaced by a random string) by the operator and

we never had the direct access to the actual mobile user ID. Second, predicting socio-economic levels of urban regions is of great importance for government. These data are used for the public problem solving benefiting the city management, not for the commercial purposes or personal purposes. Third, in our study, all the data has been aggregated into different regions instead of using individual information. We focus on the characters of the city, and the results do not reflect any user's preference, which indicates the privacy of individuals cannot be leaked.

Last but not least, all of the individual data is stored in the operator's servers, with access limited to only authorized members of the research team bound by strict non-disclosure agreements. We preprocess the data under their overseeing and only take the aggregated results back for analysis. Our research has been reviewed and approved by both the operator and our local university institutional board.

C. GDP data

Gross Domestic Product (GDP) is counted by Shanghai government once a quarter, which reflects the socio-economic level of a region. We obtain GDP of each district in 2016 from *Shanghai Economy Almanac* (2017) edited by Development Research Center of Shanghai Municipal People’s Government. Besides, we obtain the population of each block in Shanghai via the The Sixth National Census in China. We calculate the GDP of each block according to the population proportion.

The data used in our research are provided by China Telecom, which is under the agreement of protecting the data privacy. Since the raw App usage records are from individuals, we have no right to open the data with private information of users. In order to help the readers understand our research and benefit from it, we open the minimal datasets according to the *PLOS’ New Data Policy: Public Access to Data*. The minimal datasets contain all the types of data used in the research. The datasets used in this study have been deposited in the Figshare(https://figshare.com/s/792675e6995d47648c70). We confirm that these datasets that we upload could help readers and researchers to recreate the results of our paper. In order to help the readers to obtain the results and analysis with the data we provided, we would also like to open out codes at https://github.com/renxiaowo/SEL_code. Researchers who want to have access to the whole datasets can contact with Chang Liu via liu_yang@ctbri.com.cn.
